# HCV Glycoprotein Structure and Implications for B-Cell Vaccine Development

**DOI:** 10.3390/ijms21186781

**Published:** 2020-09-16

**Authors:** Luisa J. Ströh, Thomas Krey

**Affiliations:** 1 Institute of Virology, Hannover Medical School, 30625 Hannover, Germany; Stroeh.Luisa@mh-hannover.de; 2 Center of Structural and Cell Biology in Medicine, Institute of Biochemistry, University of Luebeck, 23562 Luebeck, Germany; 3 German Center for Infection Research (DZIF), Partner Site Hannover-Braunschweig, 30625 Hannover, Germany; 4 German Center for Infection Research (DZIF), Partner Site Hamburg-Luebeck-Borstel-Riems, 23562 Luebeck, Germany; 5 Excellence Cluster 2155 RESIST, Hannover Medical School, 30625 Hannover, Germany; 6 Centre for Structural Systems Biology (CSSB), 22607 Hamburg, Germany

**Keywords:** Hepatitis C virus, glycoprotein, neutralizing antibodies, nAbs, vaccine, neutralization epitope

## Abstract

Despite the approval of highly efficient direct-acting antivirals in the last decade Hepatitis C virus (HCV) remains a global health burden and the development of a vaccine would constitute an important step towards the control of HCV. The high genetic variability of the viral glycoproteins E1 and E2, which carry the main neutralizing determinants, together with their intrinsic structural flexibility, the high level of glycosylation that shields conserved neutralization epitopes and immune evasion using decoy epitopes renders the design of an efficient vaccine challenging. Recent structural and functional analyses have highlighted the role of the CD81 receptor binding site on E2, which overlaps with those neutralization epitopes within E2 that have been structurally characterized to date. This CD81 binding site consists of three distinct segments including “epitope I”, “epitope II” and the “CD81 binding loop”. In this review we summarize the structural features of the HCV glycoproteins that have been derived from X-ray structures of neutralizing and non-neutralizing antibody fragments complexed with either recombinant E2 or epitope-derived linear peptides. We focus on the current understanding how neutralizing antibodies interact with their cognate antigen, the structural features of the respective neutralization epitopes targeted by nAbs and discuss the implications for informed vaccine design.

## 1. Introduction

Chronic Hepatitis C virus (HCV) infection is one of the major causes of liver fibrosis, liver cirrhosis and hepatocellular carcinoma (HCC). Although direct-acting antivirals (DAAs) for an effective treatment reaching cure rates of > 95% are available [1], approximately 71 million people worldwide are still chronically infected with the virus and thus at risk for developing end-stage liver disease [2]. DAAs are only available to a fraction of infected patients in developing as well as in developed countries primarily due to a low awareness of the infection status resulting in a diagnosis at a late timepoint of the infection [3]. DAA treatment does not prevent cancerogenesis; and HCC recurrence rates might even increase after DAA treatment [4,5,6]. In addition reinfection remains a problem within high-risk groups [7]. Hence the development of an effective prophylactic vaccine remains a major step towards controlling and eventually eliminating HCV globally.

Several studies have demonstrated that a rapid induction of neutralizing antibodies (nAbs) along with a broadly reactive T-cell response leads to spontaneous clearance of acute HCV infections [8,9]. Vaccine candidates targeting the two HCV envelope glycoproteins, E1 and E2, have been shown to induce a strong humoral immune response in guinea pigs and chimpanzees [10,11,12] and clinical trials [13], while a broad and potent nAb response in mice appears more difficult to elicit [14]. A passive transfer of polyclonal Abs, isolated from the serum of a chronically infected patient, prior to the challenge, protects from infection with homologous HCV strains in chimpanzees and human liver-chimeric mice [15,16]. In addition, passive transfer of potent broadly nAbs (bnAbs) prior to the challenge protects from infection with heterologous viruses [17,18] and viral clearance during an acute infection leads, in about 80% of the cases, to clearance of subsequent reinfections due to a rapid induction of cross-reactive nAbs [19]. Overall, the identification of potent bnAbs that develop in the course of a natural infection has sparked hope for the development of an effective vaccine, but the scenario appears complex when compared to other viral pathogens. The extreme genetic diversity of circulating HCV genotypes [20] is a major challenge for vaccine development and antibody responses often target glycoprotein regions that have a high mutation rate [21] and/or immunodominant epitopes that serve as decoy epitopes. In addition, the extensive glycan shielding in combination with glycan shift mutations [22,23] as well as an enhanced structural flexibility of E2 [24] are thought to impact on the elicitation of a potent and broadly reactive nAb response. In general, slow Ab response rates [25] suggest a low immunogenicity of both glycoproteins but additional unknown factors may also contribute to a weak antibody response [26,27]. The timing of the nAb response seems to be critical for the outcome of the infection, i.e., viral clearance or chronic progression [28]. A factor that has rarely been taken into account is the mode of transmission, since not only cell-free virus, but also cell-to-cell transmission events might be important for transmission, and differences in DAA resistance and nAb evasion have been observed between these modes of transmission [29,30]. Eventually, also technical barriers like the limitations of the available HCV cell culture systems and animal models have to be considered for vaccine development.

Structural and biochemical data, predominantly on E2 but also on E1 and the E1E2 glycoprotein complex, in combination with immunological studies have shed light onto the possible Achilles heels of the virus. The choice of an appropriate HCV immunogen as a vaccine candidate appears to be key for precluding the emergence of viral escape mutants. In this review we aim to focus in particular on the structural characterization of E1, E2 and the E1E2 heterodimer as a basis for rational B cell vaccine design strategies.

## 2. The E1 Glycoprotein

The E1 protein contains 191 residues and is subdivided into an N-terminal domain (residues 192–239 following the numbering of the type strain H77 polyprotein), a putative fusion peptide (residues 272–285), a conserved region (residues 302–329) and C-terminal transmembrane domain (residues 350–381). The latter is essential but not sufficient for the formation of a functional E1E2 heterodimer [31,32,33]. E1 features four conserved N-linked glycosylation sites (asparagines 196, 209, 234 and 305) in most and a fifth one (asparagine 250) in certain genotypes and the presence of these glycans is important for the correct E1E2 heterodimer assembly [34].

Glycosylation contributes to correct protein folding and thereby may also influence the formation of disulfide bonds [34]. Eight cysteine residues are strictly conserved but conflicting results have been reported on their exact intra- and intermolecular disulfide bond connectivity (recently reviewed in [35]). These conflicting results may originate from dynamic changes in the disulfide bonding network of E1 during the processes of HCV entry and morphogenesis [35].

E1 is less immunogenic than E2, but two regions targeted by nAbs have been identified: residues 192–202 (numbering according to the sequence of the prototype H77 strain) are recognized by the weakly neutralizing antibody H-111 [36] and the cross-reactive nAbs IGH-526 and IGH-505 recognize residues 313–324 [37]. The latter region is predicted in silico to be highly flexible but nAb IGH-526 binds this stretch in a helical conformation as visualized by a peptide-Fab complex crystal structure [38]. A crystal structure of the N-terminal 79 amino acids of E1 revealed intertwined E1 homodimers, which were associated through several intra- and intermolecular disulfide bounds [39]. However, folding of E1 depends on the presence of E2 [40], so that the conformation of such a truncated form folded in the absence of E2 will require additional experimental validation.

## 3. The E2 Glycoprotein

The E2 glycoprotein is, similar to E1, also a type-1 transmembrane protein. E2 is responsible for virus attachment via binding to cellular receptors, including the tetraspanin CD81 [41] and the scavenger receptor class B type 1 (SR-B1) [42]. Consequently, it contains the major antigenic determinants of the virus. Neutralization epitopes are of tremendous interest for informed vaccine design and have therefore been characterized in detail in the past two decades. They predominantly overlap with the binding site for CD81 that is composed of (1) a conserved N-terminal region (residues 412–423, epitope I), (2) a front layer region comprising of a short α-helix (aa428–446) and (3) an adjacent loop named CD81-binding loop (aa519–535). Structural characterization of bNAb fragments in a complex with soluble E2 ectodomains or ectodomain fragments revealed that a number of potent cross-neutralizing bnAbs—often derived from the VH 1-69 gene family—recognize a complex conformation-sensitive epitope, called complex “front layer epitope” (CFL) [43,44,45,46]. However, not all Abs targeting one of these epitopes neutralize HCV infection, although similar contact residues are employed [47,48,49]. In addition, mutations within and outside core nAbs epitopes can confer resistance or sensitivity to neutralization [50].

### 3.1. Hypervariable Regions (HVRs)

E2 features three hypervariable regions (HVR) termed HVR1 (residues 384–411 in the prototype H77 sequence), HVR2 (residues 460–485) and the intergenotypic variable region (igVR, residues 570–580). HVR1 interacts with SR-B1 during virus entry [42] and seems to serve as an “immune decoy”. Viruses lacking the HVR1 are still infectious and the HVR1 is not critical for virus production [51,52,53]. In patients, HVR1 evolves rapidly under immune pressure during an acute infection and the accumulation of sequence substitutions continues during the chronic phase [54,55,56]. Abs directed to the HVR1 seem to be abundant [57], and can neutralize efficiently but are mostly isolate-specific [58], but the HVR1 is also targeted by non-nAbs [59]. It has been suggested that the HVR1 masks neutralization epitopes and viruses, because viruses lacking the HVR1 are more susceptible to neutralization by patient sera and nAbs [51,52]. Genotypic differences in neutralization sensitivity appear to be connected to the HVR1 and its shielding of important neutralization epitopes [60]. Additionally, the binding of poorly neutralizing Abs to HVR1 can block the binding of bnAbs to adjacent, conserved regions on E2 and hence, the induction of anti-HVR1 Abs seems to interfere with a protective humoral response against HCV infection [61]. It has been initially suggested that a ΔHVR1 E1E2 glycoprotein could be a better vaccine candidate. Indeed, sera from vaccine studies in healthy individuals display a high reactivity against the HVR1 [62]. However, a ΔHVR1 E1E2 immunogen induces a similar cross-genotype Ab response in mice and guinea pigs as the full-length E1E2, and the deletion of the HVR1 decreases serum neutralization efficiency against a homologous virus [63]. Although a full-length antigen was used in one of the vaccine groups, the majority of the anti-E2 Ab in both groups seems to target regions outside the HVR1 domain [63].

Eleven highly conserved N-linked glycosylation sites are known in E2 [64], and the removal of glycans at positions N1, N2, N4, N6 and N11 but not at positions N5 and N9 enhances nAb sensitivity [65]. However, in the absence of HVR1 glycans do not modulate neutralization sensitivity [50], leading to a model where the HVR1 and N-linked glycans of E2 cooperate to stabilize a closed E1E2 envelope conformation and thereby regulate sensitivity to nAbs [50]. The modulation of the balance between closed and open glycoprotein conformation (e.g., envelope breathing) seems to impact also on the SR-B1-dependency [50]. Indeed, the differences in neutralization sensitivity of HCV subtypes, in particular for the two highly related HCV genotype 2a strains J6 and JFH-1, could be traced back to an isolate-dependent interaction with SR-B1 and not to the presence of HVR1-specific nAbs [66]. J6, which is resistant to tested genotype 1a E1E2 antisera, binds SR-B1 directly via its HVR1 in contrast to the neutralization-sensitive JFH-1, which does not engage SR-B1. This observation gave rise to the hypothesis that the J6 virus could enter the cell faster due to the SR-B1—HVR1 interaction and thus reducing the nAbs exposure time, which consequently results in neutralization resistance [66]. Indeed, virus-associated lipoproteins can similarly increase viral infectivity through enhanced interactions with SR-B1 leading to a reduction in Ab neutralization, but also in an HVR1-dependent manner [67,68,69].

In addition to the cooperative effects, which were seen for glycans and the HVR1 [50], the presence or absence of specific glycosylation sites can influence neutralization directly. Glycans can be either essential for nAb binding [45] and directly interacting with nAbs [44] or differences in the glycosylation pattern due to glycan shift mutations can alter or abolish neutralization in other cases [70,71,72].

Similar to the HVR1, HVR2 and the igVR were found to modulate the accessibility of the CD81 binding site and the presentation of neutralizing epitopes on the E2 ectodomain [73]. Both regions are neither direct targets for nAbs nor is one of them directly involved in receptor binding but they seem to be important for structural integrity of E2 and the E1E2 heterodimer [74]. In general, a molecular dynamics (MD) simulation reveals that all three variable regions, but in particular HVR1, display very high flexibility and are likely disordered [75]. However, motions within HVR1 seem to be communicated throughout E2 to distant regions and are maintained between different viral strains [75].

### 3.2. E2 Structures

The extensive N-linked glycosylation pattern and 18 conserved cysteines forming the disulfide-bridge network of the protein [76] render the ectodomain of E2 a rather difficult target for high-resolution structural studies. Recombinantly expressed HCV glycoproteins form disulfide-bonded aggregates, and these high-molecular-weight forms elicit distinct antibody specificities with potent and broad neutralizing activity against all seven HCV genotypes [77]. In spite of these difficulties, structural information on HCV glycoproteins have been accumulated recently. The E2 ectodomain seems to retain a functional conformation when expressed without E1 in various soluble recombinant versions. The first two published crystal structures of E2 core fragments from genotype 1a and 2a, respectively, complexed either with a Fab fragment of the bnAb AR3C or a Fab fragment of the non-nAb 2A12, revealed a central immunoglobulin (Ig)-like β-sandwich with two adjacent layers, one in front and one at the back [43,78]. Larger parts of the protein are found in loop configurations or are disordered [43]. HVR1 and HVR2 were either deleted or substituted by a linker in the expression construct [78] or electron density was lacking in the respected region [43]. The igVR is not defined by electron density in the complex structures with the 2A12 Fab, in the AR3C Fab-E2 core complex structure it adopts a disulfide-constrained loop (C^569^-C^581^) [43]. More recently, Flyak and coworkers described four structures of the full-length E2 ectodomain: three from genotype 1b complexed with Fab fragments of the bnAb HEPC3, HEPC76 and AR3X respectively, and one of a genotype 1a ectodomain engaged in a ternary complex with Fab fragments of the bnAbs HEPC46 and HEPC3 [44,46]. Although the four E2 ectodomain structures are derived from different strains, they are very similar in their core region and feature common disulfide bond connectivity [44,46] (Table 1). Of note, the common disulfide bond patterns of the ectodomain structures differ from the one observed in an E2 core fragment of the genotype 1a strain in complex with the bnAb HEPC3 Fab [44]. Of note, the first two published E2 core structures themselves show variations in their respective disulfide bond network [79] (Table 1). Based on the available E2 ectodomain and core structures in particular four conserved disulfide bridges, including C^494^-C^564^, C^508^-C^552^, C^607^-C^644^ and likely also C^429^-C^503^, are critical for E2 folding (Table 1). However, it is believed, that the intra- and intermolecular disulfide bond connectivity undergoes dynamic changes during the replication cycle of the virus, e.g., upon host receptor binding [80].

While large parts of the E2 ectodomain from isolate 1b09 were not well defined in the electron density of complexes with HEPC74 Fab and AR3X Fab, the full ectodomain in the complex with HEPC3 Fab revealed structural information on (a) the intact epitope I in the context of a full E2 ectodomain and (b) the HVR2. The HVR2 encloses a flexible loop that wraps over the igVR, a scenario that is also seen in the ectodomain structure from isolate 1a53 [44]. The previously observed disulfide bridge C^569^-C^597^ constraining the igVR-loop is conserved [78] but it seems that the igVR folds further towards the front layer underneath the HVR2 in the context of an HVR2-containing ectodomain construct and an additional C^581^-C^585^ is formed, which was not observed in previous structures [44]. Epitope I (residues Q^412^ to N^423^) adopts two distinct conformations in the ectodomain structures from isolate 1a53 and 1b09 and a third conformation in the HEPC74 complex of isolate 1b09 with clear electron density only for residues G^418^ to N^423^ [44]. In contrast, clear electron density was observed for residues W^420^ to N^423^ in the AR3X complex structure, pointing in a different direction [46]. In the E2 isolate 1a53 core structure clear electron density was observed only for residues L^422^ to N^423^ [44].

**Figure 1:**
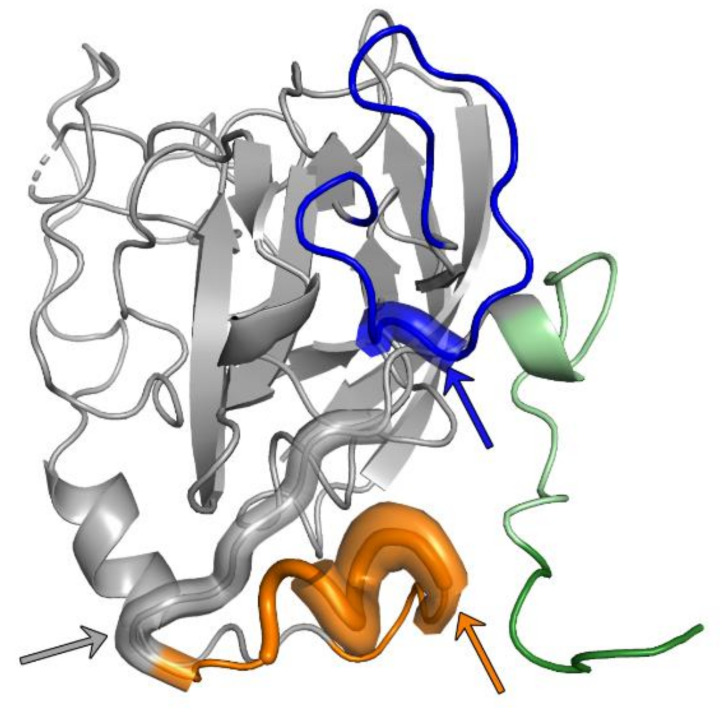
Structurally characterized neutralization epitopes within Hepatitis C virus (HCV) E2. Cartoon representation of the E2 ectodomain crystallized in a complex with HEPC3 and HEPC46 Fab (PDB 6MEJ). The Fab fragments were removed for clarity. The ordered part of the hypervariable region 1 (dark green) and the composite CD81 binding site consisting of epitope I (aa412–423; palegreen), epitope II (aa434–446; orange) and the CD81-binding loop (aa518–535; blue) are highlighted in color. A conserved frontlayer epitope (residues 427–434, 438–443 and 529–531) that is targeted by several bnAbs derived from the VH1-69 germline gene (see text) is indicated by wider and transparent cartoon segments and labeled by colored arrows.

Three additional structures of an E2 core fragment (with HVR1, HVR2 and IgVR deletions) from genotype 6 in a complex with AR3C, AR3B or AR3D Fab, respectively, are highly similar within their core regions compared to the previously described E2 core structures [45]. Minor conformational differences were observed in the β-sandwich loop connecting β6 and β7 (residues 540–552) in comparison to the E2 genotype 1—AR3C Fab complex [43,45], suggesting that a substitution at position 540 that eliminates an N-glycosylation site causes these differences [45]. Interestingly, similar conformational variations within the β-sandwich loop are observed between the genotype 1b full-length E2 ectodomain complexed either with HEPC3 or HEP74 [44]. These results indicate that the loop connecting β6 and β7 of the β-sandwich likely also adopts different conformations in the context of the HCV virion, underlining the observed conformational flexibility of E2 [24,81] (Figure 1).

### 3.3. Epitope I

Synthetic E2 peptides of epitope I and II were complexed with Fabs or scFv fragments from nAbs and non-nAbs (reviewed in detail also in [24] and [81]) in several additional crystallographic studies. For epitope I, a very similar β-hairpin conformation with only small conformational differences was observed for the human nAb HCV1, the murine nAb AP33, the humanized and affinity-matured nAbs MRCT10.v362 and hu5B3.v3 (derived from AP33 and mu5B3, respectively) and the murine nAb MAb24 [82,83,84,85,86]. However, AP33 and HCV1 Fab fragments seem to engage the epitope I on E2 from different directions [82]. In contrast, rat nAb 3/11 recognizes the same peptide in an extended conformation [87]. A cluster of human nAbs named HC33 engages residues I^414^ and N^415^ in a β-sheet conformation and the C-terminal part of the peptide is recognized in an extended conformation [71]. The resulting turn (residues T^416^ to S^419^) superimposes well with the turn observed in the β-hairpin conformation in complex with HCV1 and AP33 Fabs. The obtained structures of epitope I-peptides in a complex with bnAb fragments differ from the conformation observed in the context of the whole E2 ectodomain [44], and therefore likely represent individual snapshots, indicating that the epitope is either present in different conformations on the HCV particle, or can be induced into different conformations depending on the interaction with individual nAbs. This conformational flexibility was also visualized by electron microscopy demonstrating that the HCV1 Fab binds a soluble E2 from different angles of approach [88]. Dose-dependent neutralization effects and similar neutralization potencies of epitope I—specific nAbs suggest a dynamic equilibrium between the different conformations, which can be converted easily in either direction following an “induced-fit” nAb binding mode [87]. However, MD simulations suggest that the different conformations rather represent captures of transient conformations [75]. Genotype-specific sequence differences seem not to dictate the predominant epitope I-conformation but nAb neutralization efficiencies may be still modulated by distinct sequence variations [87]. The structural flexibility within epitope I could explain the modest immunogenicity of this epitope in HCV-infected patients [89].

### 3.4. Epitope II

Similar structural snapshots using Fabs and synthetic E2 peptides have been reported for epitope II (residues 434–446). When engaged by potent nAbs HC84.1, HC84.27 and the affinity-maturated nAb HC84.26.5D, residues W^437^ to F^442^ form a short α-helix with an extended conformation on the C-terminal side comprising residues 443–446 [90]. This short α-helix can also be found in the E2 ectodomain and core complex structures but minor differences in the spatial arrangement of the C-terminal part of epitope II (aa443-aa446) [91] suggest that local structural changes may occur. The non-nAb #12 and the weakly neutralizing antibody #8 recognize a similar epitope including the short α-helix and a few amino acids upstream [49,92]. However, the two residues W^437^ and L^438^ crucial for binding of these two murine Abs are neither solvent-accessible in the E2 core [43,44,45] nor in the full-length ectodomain structures [44], suggesting that a conformational change exposing these two residues is required to allow E2 binding of Abs #8 and #12 [49,88]. In contrast, the epitopes of the potent nAbs HC84.1, HC84.26.5D and HC84.27 are solvent-accessible in complexes with AR3-nAbs [43,45], HEPC3 and HEPC74 [44], indicating that this latter “closed” E2 state represents a preferred conformation in the HCV particle.

### 3.5. The CD81-Binding Loop

In the E2 structures complex with potent bnAbs recognizing the front layer epitope the CD81-binding loop together with the entire front layer is stabilized by the engaging Fab. The side chains of F^537^ and L^539^, located in β-strand E of the Ig-like domain, are buried in these structures inside the hydrophobic core of the Ig-like domain (closed conformation) [43,44,45]. In contrast, a helical conformation, in which residues F^537^ and L^539^ are buried in the Fab interface, is observed in the crystal structure of the DAO5 Fab in a complex with the E2 peptide comprising of aa532–540 [48] (open conformation). More evidence in support of such a conformational flexibility also in the Ig-like domain comes from a structure of an E2 core fragment in complex with a non-nAb targeting a different antigenic region, in which residues 524–535 are disordered and F^537^ is solvent-exposed in the absence of stabilizing Fab interactions with the front layer epitope [78]. The open and close conformations can be found simultaneously on infectious particles [48]. This observation lead to the hypothesis that the open conformation or intermediate conformational states may act as immunological decoys distracting the humoral immune system from the relevant CD81-binding conformation [48].

### 3.6. The Front Layer Epitope

Several bnAbs have been identified that recognize a conserved region located in the E2 front layer named front layer epitope [43,44,45,46]. Indeed, the potent bnAbs HEPC3 and HEPC74, isolated from individuals who spontaneously cleared HCV infection [44,93], share a common sequence signature with the earlier described bnAb AR3C [44]. These three bnAbs are derived from the same VH1-69 gene, which has been frequently found in human bnAbs targeting different viral glycoproteins [44,90,93,94]. A structural superposition of E2 reveals that the complexed HEPC3 and HEPC74 Fabs were rotated by about 89 and 77°, respectively, relative to the bound AR3C Fab. Thereby, interactions between the tips from HEPC3, HEPC74 and AR3C CDRH3 loops with the same conserved residues in the front layer of E2 are possible, although HEPC3 and HEPC74 differ in their CDRH3 loop configuration from AR3C. The two cysteines, forming a disulfide bond in the CDRH3 regions of all three antibodies, are derived from the human germline D segment 15 (IGHD2–15) in the case of HEPC3 and HEPC74 or encoded by either IGHD2–21 or IGHD2–15 in the case of AR3C. The disulfide motif is important for E2 binding and neutralization, and has been suggested to play a critical role for initial germline recognition [44]. Hence the front layer epitope represents a promising candidate for targeted immunogen design.

## 4. The E1E2 Heterodimer

The current model of HCV particles suggests that E1 and E2 are organized as trimers of E1E2 heterodimers on the surface of the infectious virion [95]. However, a complete structural picture of the E1E2 heterodimer is still missing due to an incomplete biochemical characterization in the context of infectious virions and difficulties in the expression of a recombinant, natively folded heterodimer. In the absence of a high-resolution structure biologically validated computational models [96,97,98] and a low-resolution TEM reconstruction [99] provide first insights into E1E2 heterodimerization. A comparison of the computational models reveals similarities, especially in the E2 core, but also major differences, concerning the relative orientation of E1 to E2, the presence of an intermolecular disulfide bond, and the conformation and orientation of HVR1 (reviewed in [100]).

Intracellular forms of E1 and E2 were reported to be assembled as non-covalent heterodimers, but covalent dimers and oligomers stabilized by disulfide bonds are found on infectious viral particles. It remains elusive to date whether such high molecular weight glycoprotein forms are actually required for function [101]. A deletion of the glycoprotein’s transmembrane domains (TMs) or its replacement by the anchor signal of another protein abolishes the formation of the E1E2 heterodimer [32], underlining that the TMs play a major role in the assembly of the E1E2 heterodimer. Protein folding and maturation processes of the two glycoproteins depend on the presence of the other glycoprotein and especially the folding of E1 and the potential formation of E1 trimers depends on the presence of E2 and a specific G_354_xxxG_358_ motif located within the E1 TM [95]. However, the expression of E1 and E2 *in trans*, i.e., from two different cistrons, is sufficient for the heterodimerization in the ER, suggesting that expression *in cis* is not required. Additional residues within E1 (such as residues 308, 330 and 345) are important for the functional interaction between E1 and E2 [33] and MD simulations have proposed a key role of the K_370_-D_728_ salt bridge [102,103] for transmembrane heterodimerization. Mutagenesis experiments led to the hypothesis that the N-terminus of E1 is associated with the C-terminus of E2 [17].

To facilitate structural, biochemical and immunological studies different expression systems including yeast [104], insect cells [105], mammalian cells [12,106] or *Leishmania* [107] have been explored to obtain intact recombinant E1E2 heterodimers as well as soluble truncated forms. E1E2 glycoproteins can be purified from Chinese hamster ovary (CHO) cells as membrane-associated complexes using either lectin purification or an Fc-affinity tag inserted in the junction between E1 and E2 [12,14].

In an early approach, a non-glycosylated E1E2 chimeric protein containing the two linked ectodomains was purified via refolding methods from bacteria [108]. Binding to CD81 was confirmed and antibody titers induced in rabbits were higher for the chimera than E1 or E2 alone but further functional information were not reported [108]. In a different study, the E1 and the E2 ectodomains were connected by a small hydrophilic peptide (eight residues; FLAG-tag) for expression using the baculovirus system [105]. A higher yield was described for a permuted fusion protein, with an N-terminal E2 ectodomain linked via a TEV protease to the E1 ectodomain [109]. Both fusion proteins were observed as a monomeric species but had a high tendency to self-associate to higher oligomers. Their overall secondary structure seemed similar in containing a large non-structured region [109].

Several different linkers between E1 and E2 replacing the E1 transmembrane domain for soluble E1E2 production were tested using a panel of E1, E2 and E1E2 specific Abs [110], but a soluble E1E2 complex (sE1E2) could still be obtained by simply deleting the transmembrane domains without the addition of a flexible linker. A linker containing a protease cleavage site and a Strep-tag connecting the E1 and E2 ectodomains yielded a soluble E1E2 molecule (sE1E2v4), which was recognized in a similar manner by conformational E1E2-specific nAbs. However, further analysis showed a high percentage of oligomers in the purified material [110]. Subsequent immunization of mice revealed, that the sE1E2 molecule elicited higher Ab titers and greater reactivity breadth than cell-associated E1E2 but weak neutralizing activity was elicited by both immunogens. The overall level of serum nAbs was insufficient for neutralization although detected nAbs were specific for conserved E2 antigenic sites [110]. Nevertheless, tested constructs could serve as a template for more detailed structural studies of the ectodomain and rational antigen design. Hence, in order to obtain E1E2 heterodimer ectodomains for electron microscopy studies earlier approaches were picked up and modified leading to a suitable soluble heterodimer with either an IgG Fc fragment (Flag- and His_6_-tagged) or a de novo designed dimerization-tag expressed in insect cells [99]. Both heterodimer constructs were obtained as higher oligomers but also as monomers with the latter ones yielding monodisperse particles on negative stain grids. sE1E2-Fc particles feature a head and a tail region in the 2D averaged classes with the head region corresponding to the E1E2 heterodimer and the tail region formed by the Fc homodimer [99]. Similarly, 2D averaging for negative stain images collected for the second construct revealed a doughnut-shaped head and a tail, which correspond to the E1E2 heterodimer and the dimerization tag, respectively. A comparison of the recombinant monomeric E1E2 heterodimer under reducing and non-reducing conditions on the SDS-PAGE gel suggests that one or more intermolecular disulfide bonds are formed, which is consistent with virion-associated E1 and E2 [101]. The heterodimer constructs were also used for expression in mammalian cells with a similar experimental outcome although an increased heterogeneity of the purified sample likely due to larger and rather inhomogeneous glycosylation was observed [99]. Interestingly, monomers as well as higher oligomers were bound by conformational E1 and E2-specific nAbs as well as CD81, suggesting that both species contain at least partially correctly exposed epitopes and a functional receptor binding site. Furthermore, the soluble E1E2 heterodimer could inhibit HCV infection. A 3D reconstruction for the de novo dimerization-tagged heterodimer at 27 Å resolution highlighted two density blobs likely corresponding to the E1 and E2 ectodomains [99]. Docking of the available crystal structures of the N-terminus of E1 [39] and the E2 core [43,78] was difficult due to missing segments and the low resolution of the electron density map. Hence, a coevolution analysis approach and structure prediction by Rosetta were applied [99]. The resulting model suggests that both ectodomains can be divided into an N-terminal region and a stem region forming thereby two interfaces. One of them is located at the membrane distal ends of the N-terminal regions and the other one is located near the membrane proximal ends of the stem regions [99]. The putative fusion peptide of E1 forms a helix in the presented model and interacts directly with E2, but Rosetta modeling of E1 suggests also several alternative conformations for the fusion peptide [99]. Deletion mutants at the proposed interface between E1 and E2 did abolish the production of soluble protein [99].

## 5. Implications for a Structure-Based Design of a B Cell Vaccine

During the initial stages of the infection including initial attachment and receptor engagement, the glycoprotein complex is likely to be exposed to circulating nAbs. Hence, targeting these early receptor interactions is the most likely route to an effective B-cell vaccine. The accumulating structural information on HCV glycoproteins suggest that the design of suitable immunogens will be key for success. So far, several types of B-cell vaccine strategies have been explored in combination with adjuvants including recombinant E1E2, E1 and E2 glycoproteins, DNA vaccines expressing these envelope proteins, chimeric HBV-HCV envelope proteins and virus-like particles (VLPs) [111,112,113,114,115]. Administration of a subunit vaccine consisting of the CHO-cell expressed E1E2 glycoproteins from genotype 1a elicited a human antibody response mainly directed against the HVR1 and was rather isolate-specific [12,62]. Accordingly, it has been proposed that conserved viral epitopes could be made considerably more accessible for nAb binding by removal of the HVR1. Unexpectedly, an E2 lacking HVR1 and selected glycosylation sites was unable to elicit cross-nAbs in mice suggesting that exposure of conserved epitopes through deletion of potentially interfering regions is not sufficient to focus the Ab responses on the production of cross-nAbs [116]. A similar observation was also reported for the E1E2 glycoprotein comparing a wild type and a ΔHVR1 version and the removal of HVR1 adversely affects the immunogenicity of the glycoprotein [63]. The HVR1 may influence the conformation of the glycoproteins or its conformational flexibility and, subsequently, the exposure of neutralizing epitopes [50,66], suggesting that further immunogenic engineering besides the HVR1 deletion is required to generate an improved immunogen. A recombinant E2 version lacking all three hypervariable regions retains CD81 receptor binding and refocuses the antibody response onto epitope I and CFL-like specificities, albeit it elicits low Ab titers [77].

Recent studies on other viruses such as HIV have shown that further protein engineering including the introduction of disulfide bonds can improve the stability of a certain conformation and hence the antigenicity [117]. In addition, a certain amino acid substitution within the engineered HIV SOSIP gp140 trimer limits the transient exposure of non-neutralizing, immunodominant epitopes, which are proposed to interfere with the induction of bnAbs [118]. Residues within but also outside non-neutralizing epitopes can be critical for the immunogen design.

Interestingly, considerable variations in neutralization sensitivities to nAbs were detected in a broad study using a large panel of HCV pseudoparticles (HCVpp) and infectious cell-culture derived particles (HCVcc) [119,120]. These differences are unrelated to the genotype and have been suggested to originate from isolate-specific polymorphisms [119]. Indeed mutations within and outside neutralization epitopes can confer resistance or sensitivity to neutralization by bnAbs. Similarly, a panel of HCVpps comprising of the E1E2 glycoprotein from genotype 1 isolates was characterized according to neutralizing breadth and resistance to 18 previously described human bnAbs [121]. A detailed sequence analysis reveals that polymorphisms at non-contact residues may constitute a major immune evasion mechanism facilitating viral persistence and have to be taken in consideration for vaccine development [121].

In cases, where conventional vaccines failed to raise an immune response and immunization with only synthetic peptides alone is not successful, the backbone of the immunogen epitope can be conformationally confined, either by epitope-focused immunogen design or by chemical peptide modification, e.g., peptide cyclization. [122,123,124]. Such a stabilization of the immunogenic conformation is able to improve antigenicity and results from other viruses such as respiratory syncytial virus (RSV) [122] and Influenza [125] have stimulated similar studies also on HCV neutralization epitopes [126,127,128].

Immunization of mice with a cyclic variant of epitope I (c-Epitope I) elicited Abs that bind E2 with high affinity but failed to neutralize. Structural characterization of a c-Epitope I—Fab complex revealed that residues W^420^ and glycosylated N^417^, which are solvent exposed in other complexes, are largely buried and the cyclic peptide is rotated by 180° at the complex interface compared to the previously observed orientations [127]. It was suggested that due to the high selectivity for the cyclic epitope, elicited Abs fail to interact with epitope I in a more flexible and extended conformations as present on the virus. Auspiciously, a cyclic defensin design and a bivalent immunogen with two copies of the epitope on the E2 surface induced greater epitope-specific responses in mice and enhanced serum neutralization efficiency compared to the native peptide epitope [128]. However, the neutralization breath against heterologous HCVpps in particular in the case of the cyclic defensin constructs was limited [128], suggesting that despite robust epitope I-specific responses, other factors may still reduce the heterologous neutralization capacity of immunized sera [128]. In another study, He and coworkers identified ten E1 (antigenic site 314–324) and ten E2 epitope I scaffolds for further computational design and experimental validation [126]. Three E1 and five E2 scaffolds were positive for binding to nAbs IGH526 and HCV1, respectively [126]. Different binding kinetics were observed for the identified scaffolds but immunization data for these new immunogens are not yet available [126]. In an alternative approach, an anti-idiotypic Ab, which also functions by mimicking a neutralization epitope on an unrelated protein (in this case an Ab), was demonstrated to robustly induce HCVcc-nAbs [129], suggesting that epitope-focused immunogens represent a viable strategy to develop a safe and efficient B cell vaccine and elicit a protective nAb response.

It is well established that nanoparticle presentation of viral glycoproteins improves (humoral) immune responses and similar approaches have been explored for HCV (reviewed in [130]). Interestingly, higher order oligomers of recombinantly expressed E2 also improves humoral immune responses [77], suggesting that multivalent presentation of conserved E2 epitopes works in both nanoparticles and higher order glycoprotein aggregates. A DNA vaccination approach, in which secreted E1 and E2 are incorporated into oligomers by fusion with the oligomerization domain of the C4b-binding protein, elicits promising nAb-responses in vaccinated mice [131]. VLPs, which self-assemble into non-infectious particles, also represent a safe and immunogenic vaccine delivery platform. However, to date VLP-based HCV vaccines have not progressed into clinical trials although promising initial results have been reported [130].

Self-assembled core-E1-E2 HCV-like particles (HCV-LPs) were found to induce strong and broad polyclonal cellular and humoral immunity [132,133]. The insertion of full-length E1, E2 or E1E2 into Hepatitis B virus (HBV)-based VLPs via the surface antigen (HBsAg-S) VLPs yielded chimeric HBV-HCV particles similar in size and shape to the wildtype HBsAg subviral particles [134]. Immunization of rabbits with these chimeric HBV-HCV particles resulted in elicitation of cross nAbs against HCVpp genotypes 1a, 1b, 2a and 3 [114,135]. Interestingly, humoral responses observed in animals vaccinated with E1E2-containing HBsAg VLPs were reduced compared to HBsAg VLPs expressing both proteins separately [135], suggesting that the E1E2 heterodimer is less immunogenic, possibly due to masking of immunodominant epitopes. HBV-HCV chimeric particles also induced significant levels of HBV-specific nAbs, supporting the idea of a bivalent vaccine [135]. Another HBV-related immunogen platform, the HBV core protein (HBc) assembles into dimers and subsequently into VLPs also in the presence of insertions of foreign sequences into the gene. Up to now, this platform has been used for NS3 and HCV core gene segments [136,137,138], and although subsequent immunization in mice yielded high levels of anti-NS3 Abs only relatively low antibody titers to HCV core epitopes were detected [136,137]. The multimeric papaya mosaic vaccine platform has also been used to present an HCV E2 peptide (residues 511–530) and triggered a strong humoral immune response in mice [139], confirming that the multivalent presentation of the antigen improves its immunogenicity. Multivalency has been combined with the epitope-based scaffold design for E1 aa314–324 and the E2 epitope I, displaying 24 copies of the respective epitope scaffold on ferritin, a robust self-assembling protein from *Helicobacter pylori*, but humoral responses from animal experiments have not yet been reported [126]. Similarly, soluble E2 has been engineered on self-assembling ferritin particles, resulting in an increased potency to elicit neutralizing antibodies when compared to soluble E2 in its monomeric form [140].

## 6. Conclusions

The multiple viral evasion mechanisms that preclude immunogenicity of the conserved neutralization epitopes within HCV E2 (e.g., genetic variability, conformational flexibility, decoy epitopes and epitope shielding by glycans and lipoproteins) suggest that soluble glycoproteins alone induce a polyclonal antibody response of insufficient potency and breadth. The data reported to date point out the necessity to use novel strategies of immunogen design that overcome the described evasion mechanisms. One of these strategies is certainly a structure-based protein engineering in combination with multivalent epitope presentation in nanoparticles, as this approach has been demonstrated to drastically increase the induced neutralization titers for other RNA viruses like the respiratory syncytial virus (RSV) [141]. Such improved B cell immunogens can be combined with recently improved replication-competent viral vectors that have not been discussed in this review, thereby inducing both a strong T cell response and a potent antibody response and paving the way for a safe and efficient HCV vaccine.

## Figures and Tables

**Table 1:** Disulfide bridge network present in the E2 core and ectodomain structures.

	Disulfide Bond	E2 Core 1a53	E2 Core H77	E2 Core J6	E2 Core GT6a	E2 Core GT6a	E2 Core GT6a	E2 Ecto 1b09	E2 Ecto 1b09	E2 Ecto 1a53	E2 Ecto 1b09
PDB		6MEK	4MWF	4WEB	6BKD	6BKB	6BKC	6MEI	6MEH	6MEJ	6URH
Complexed Fab		HEPC3, HEPC46	AR3C	2A12	AR3D	AR3A	AR3B	HEPC3	HEPC74	HEPC3, HEPC46	AR3X
	429–503	+	+	§ C429	+	+	+	+	+	+	+
	452–620	+	+	-	disordered	+	disordered	+	+	+	+
	459–486	disordered	disordered	-	disordered	disordered	disordered	+	+	+	disordered
	486–620	-	-	+	disordered	disordered	disordered	-	-	-	disordered
	**494–564**	**+**	**+**	**+**	**+**	**+**	**+**	**+**	**+**	**+**	**+**
	**508–552**	**+**	**+**	**+**	**+**	**+**	**+**	**+**	**+**	**+**	**+**
	569–581	-	+	-	§	§	§	-	-	-	-
	569–597	+	-	+	§	§	§	+	+	+	+
	581–585	-	-	disordered	§	§	§	+	+	+	disordered
	585–597	-	+	-	§	§	§	-	-	-	-
	**607–644**	**+**	**+**	**+**	**+**	**+**	**+**	**+**	**+**	**+**	**+**
Reference		[44]	[43]	[78]	[45]	[45]	[45]	[44]	[44]	[44]	[46]

+ disulfide bridge present, - disulfide bridge absent, § not present in the expression construct, **bold**—disulfide bridge observed in all structures.
